# Supplementary material for: Antifungal activity of volatile compounds generated by endophytic fungi Sarocladium brachiariae HND5 against Fusarium oxysporum f. sp. cubense
Source: PLoS One. 2021 Dec 2;16(12):e0260747. doi: 10.1371/journal.pone.0260747 (PMC8639089; doi:10.1371/journal.pone.0260747)
Supplement: S4 Table — (DOCX) [file pone.0260747.s004.docx]

**S4 Table Primers used in this study**

| Primer | Sequence (5’-3’) | Notes |
| --- | --- | --- |
| Actin-F | GTTGGACTTGGGGTTGATGGG | qRT-PCR primers for amplification  of the reference gene *actin* |
| Actin-R | CAAGCGTGGTATTCTCACTCTGC |  |
| FOIG_00580-F | CGCTCGTTCTCATTCTTTCAGTT | qRT-PCR primers for analysis of gene (FOIG_00580) expression |
| FOIG_00580-R | CTTGTTTCGTTTCCTACGGTCAG |  |
| FOIG_06735-F | ACCGTTCCTCCGATGCGTTAC | qRT-PCR primers for analysis of gene (FOIG_06735) expression |
| FOIG_06735-R | GCAATCCGTTCTCAGTGTCAATAC |  |
| FOIG_06738-F | CATCCCAGGTGCCACAGACT | qRT-PCR primers for analysis of gene (FOIG_06738) expression |
| FOIG_06738-R | CTGACAGCGGGTGGAGTTTC |  |
| FUB2-F | CCACAGCACTGCCGAAAATG | qRT-PCR primers for analysis of *FUB2* expression |
| FUB2-R | TGACGAAGAAGCCGTGAGACA |  |
| FUB5-F | TGCTACATCGCCCTCACCAAC | qRT-PCR primers for analysis of *FUB5* expression |
| FUB5-R | CACAAGCGTAGGCTGCTCAAT |  |
