# Supplementary material for: Antifungal activity of volatile compounds generated by endophytic fungi Sarocladium brachiariae HND5 against Fusarium oxysporum f. sp. cubense
Source: PLoS One. 2021 Dec 2;16(12):e0260747. doi: 10.1371/journal.pone.0260747 (PMC8639089; doi:10.1371/journal.pone.0260747)
Supplement: Attachment — Submitted filename: response to reviewer.docx [file pone.0260747.s006.docx]

Dear Editor,

Thank you for serving as the editor of our manuscript and managing the review process. We also acknowledge the efforts of the reviewers that you assembled and appreciate their constructive comments and suggestions to improve the quality of our manuscript. We have made the suggested corrections, which are provided in the revised manuscript and specific responses to each of the reviewer’s comments can be found below. Thank you for your further consideration of our revised manuscript.

**Detail response:
Reviewer 1:** The authors have presented here the possibility of employing the volatile organic compounds from endophytic fungus Sarocladium brachiariae HND5 to antagonise the pathogen Fusarium oxysporum f. sp. Cubense of banana plants. The proper experimental approach has been carried out to support the findings.

There are few suggestions:

The introduction of the manuscript can be improved and made more informative for the reader. Introduction should reflect the extent of work that is carried out. Such as there is no mention of Fusaric acid and its role in disease development in the introduction portion to reflect the need to study its production.

**Answer:** Thank you for your constructive criticism and detailed review of this manuscript. We have added description of fusaric acid in introduction portion.

The name of the fungus Sarocladium brachiariae has to be italicised in the manuscript. Also scientific names in some other places has to be italicised.

**Answer:** We have checked all thquanrough the manuscript and changed all scientific names to italic.

The following lines from Introduction portion (pg 7 and 8) are repeated:

This pathogen can infect banana from root and invade the xylem vessels and eventually cause a lethal wilting of the infected plants [3]. This pathogen can infect banana plants from the root and invade the xylem vessels, eventually causing lethal wilting of the infected plant2.

Such repetitions of statements are in end of Discussion and Conclusion section too (pg 20 and 21): ‘VOCs it generates show promise for use as biological control agents or fumigants against FOC in agricultural production systems.’ It should be avoided.

**Answer:** We have deleted the repeated sentences.

The line in the Material and Methods is a repeat from introduction: The antagonistic strain HND5, which was isolated from healthy leaf of Brachiaria brizantha, was identified as Sarocladium brachiariae. (China General Microbiology Culture Collection Center accession No. 2192) according to the LSU and ITS rDNA sequence [30, 31].

It needs to be restructured according to the Material and methods section.

**Answer:** This sentence has been restructured according to the Material and methods section.

There are certain grammatical mistakes as hyphen has been used in between some of the words such as effec-tive, ac-id etc. in the Abstract.

**Answer:** We have checked all through the manuscript and deleted all misused hyphens.

**Reviewer 2:** A quality of work has been done by authors and well written manuscript except few mistakes done.

Firstly, add name of fungi in the title of manuscript instead of only writing HND5,

**Answer:** Thank you for your comments. They are very helpful for improving our manuscript. The fungi name has been added in the title of manuscript.

Remove hyphen wherever not required as yellow highlighted,

**Answer:** We have checked all through the manuscript and deleted all misused hyphens.

Write fungi name in italics,
**Answer:** We have checked all through the manuscript and changed all scientific names to italic.

Remove repeated sentance,

**Answer:** We have removed repeated sentences in the introduction part.

Write incubated/ion instead of inoculated/ion wherever required,

**Answer:** We have changed inoculated into incubated.

Write "parafilm/ petri" in small letters,

**Answer:** We have checked all through the manuscript and changed "parafilm/ petri" into small letters.

Write PBS, 2M4V, 34D, B-C in bracket when first appear in text in full form,

**Answer:** We have added full name of PBS, 2M4V, 34D, B-C in the text where first apper.

There are some minor speeling errors like regent, unite which should be corrected to reagent, unit,

**Answer:** We have checked all through the manuscript and corrected these spelling mistakes.

Its better to use "C" for control instead of "CK"

Answer: As many academic papers using “CK” for control, we keep “CK” in revised manuscript. Thanks for your suggestion!
