# Supplementary material for: Antifungal activity of volatile compounds generated by endophytic fungi Sarocladium brachiariae HND5 against Fusarium oxysporum f. sp. cubense
Source: PLoS One. 2021 Dec 2;16(12):e0260747. doi: 10.1371/journal.pone.0260747 (PMC8639089; doi:10.1371/journal.pone.0260747)
Supplement: Attachment — Submitted filename: renamed_5553a.docx [file pone.0260747.s007.docx]

Dear Editor,

Thank you for serving as the editor of our manuscript and managing the review process. We also acknowledge the efforts of the reviewers that you assembled and appreciate their constructive comments and suggestions to improve the quality of our manuscript. We have reviewed reference list and did not find retracted papers. Also, we have used PACE to adjust figures and uploaded adjusted figures in the revised manuscript. Thank you for your further consideration of our revised manuscript.

**Detail response:**

**Reviewer 2:**

**Title (Page 1):** Full name of fungi (Sarocladium brachiariae) have been inserted.

**Abstract (Page 1):** Misused hyphens have been removed. And fungi name have been written in italics.

**Introduction (Page 2):** We have removed repeated sentences in the introduction part.

**Introduction (Page 2):** We have checked and make sure is “low target specificity”.

**Materials and Methods (Page 4)**: We have changed "parafilm/ petri" into small letters and corrected “inoculate” to “incubate”.

**Materials and Methods (Page 5)**: We have changed "parafilm/ petri" into small letters and corrected “inoculate” to “incubate”. And we also written PBS in text in full form.

**Materials and Methods (Page 6)**: We have changed "parafilm/ petri" into small letters and corrected “inoculate” to “incubate”. And we also written 2M4V, 34D, B-C in text in full form.

**Results (Page 7)**: We have changed "parafilm/ petri" into small letters, corrected “inoculate” to “incubate” and removed misused hyphen.

**Results (Page 8)**: We have changed "regent" into “reagent”.

**Results (Page 9)**: We have changed "unite" into “unit” and corrected “inoculate” to “incubate”.

**Results (Page 11)**: We have removed misused hyphen.

**Figures:** As many academic papers using “CK” for control, we keep “CK” in revised manuscript. Thanks for your suggestion!
